# Suppression of Glial Activation in Tau Transgenic Mice Through Inhibition of CRMP2 Phosphorylation: a Morphometric Analysis

**DOI:** 10.1007/s12017-025-08891-9

**Published:** 2025-10-14

**Authors:** Wanying Li, Toshiki Kubota, Valeria Ayala Guevara, Yoshio Goshima, Takaomi C. Saido, Toshio Ohshima

**Affiliations:** 1 Laboratory for Molecular Brain Science, Department of Life Science and Medical Bioscience, Waseda University, 2-2 Wakamatsu-Cho, Shinjuku-Ku, Tokyo, 162-8480 Japan; 2 Department of Molecular Pharmacology and Neurobiology, Yokohama City University Graduate School of Medicine, Yokohama, 236-0004 Japan; 3 Laboratory for Proteolytic Neuroscience, RIKEN Center for Brain Science, 2-1 Hirosawa, Wako-Shi, Saitama 351-0198 Japan

**Keywords:** Alzheimer’s disease, Tau, Microglia, Astrocyte

## Abstract

Tauopathies, including Alzheimer’s disease (AD), are neurodegenerative diseases characterized by abnormal tau aggregation in neurons and glial cells. Various tauopathy mouse models have been developed, including PS19, a tau-overexpressing transgenic mouse model with the P301S mutation, in which glial activation has been reported prior to the accumulation of tau. In this mouse model, tau pathology was improved by the administration of the immunosuppressant FKB506, suggesting that inflammatory responses promote the progression of tau pathology. Our previous studies have shown that the inhibition of Collapsin response mediator protein 2 (CRMP2) phosphorylation suppresses inflammation and ameliorates pathological progression in spinal cord injury and MPTP-induced Parkinson’s disease models using CRMP2KI/KI mice, in which the phosphorylation site Ser522 was replaced with Ala. Therefore, we compared glial cell activation in male PS19 and PS19; CRMP2KI/KI mice using morphometric analysis at 5 months of age, before the onset of tau pathology. We found that the morphological changes caused by the activation of microglia and astrocytes were normalized by suppressing CRMP2 phosphorylation compared with those in PS19 mice. Cox-2 expression in hippocampal neurons was increased in PS19 mice, but this increase was suppressed in PS19; CRMP2KI/KI mice, suggesting that the suppression of CRMP2 phosphorylation in neurons is also involved in this process. These results suggest that the inhibition of CRMP2 phosphorylation may improve neuroinflammation in tauopathy.

**Supplementary Information:**

The online version contains supplementary material available at 10.1007/s12017-025-08891-9.

## Introduction

Alzheimer’s disease (AD) is a multifaceted neurodegenerative disorder characterized by extracellular amyloid-β (Aβ) plaques, intracellular neurofibrillary tangles of hyperphosphorylated tau protein, and a persistent neuroinflammatory state. Aβ aggregates and hyperphosphorylated tau activate the NLRP3 inflammasome in microglia, initiating a neuroinflammatory cascade (McGroarty et al., 2025).

Collapsin response mediator protein 2 (CRMP2) is phosphorylated by Cdk5 and GSK3. In patients with AD and some AD mouse models, CRMP2 phosphorylation levels are abnormally high in cerebral cortex and hippocampus and can be detected before the formation of amyloid plaques and neurofibrillary tangles, suggesting that it is an early event in AD pathogenesis (Cole et al., 2007).

We recently reported that Tau Tg; CRMP2 KI mice display significantly less microglial activation following Aβ25–35 injection than their Tau Tg counterparts (Noguchi et al., 2025). Although many factors can influence microglial and astrocyte activation, the direct impact of CRMP2 phosphorylation inhibition on glial behavior and subsequent neuronal inflammation has not yet been fully defined. To address this, the present study systematically compared microglial and astrocyte density, morphology, and inflammatory marker expression in the dorsal hippocampus of four groups: wild-type (WT), CRMP2 KI/KI, Tau Tg, and Tau Tg; CRMP2 KI/KI mice, to clarify how blocking CRMP2 phosphorylation modulates neuroinflammation.

## Materials and Methods

### Animals

All animal experiments were conducted in compliance with protocols approved by the Institutional Animal Care and Use Committee of Waseda University (Approval No. 2021-A022, 2022-A032, and A23-051). The mice were housed under standard laboratory conditions with a 12 h light/dark cycle and had ad libitum access to food and water supply. Information about mouse strains used in this study is provide in Supplemental Information.

### Immunofluorescence and Image analysis

Detailed procedure of Immunofluorescence and Image analysis is provided in Supplemental Information.

### Statistical Analysis

Methods of statistical analyses are provided in Supplemental Information.

## Results

### Attenuated Activation Trends in Microglial Dynamics and Morphology

To examine the degree of microglial activation, we immunostained the tissues of WT, CRMP2 KI/KI, Tau Tg, and Tau Tg; CRMP2 KI/KI mice using anti-Iba1 as a microglial marker. After capturing images of coronal sections from the dorsal hippocampus, we evaluated the density of Iba1-positive cells (Figure S1A). Tau Tg mice exhibited an increase in microglial density compared to WT and CRMP2 KI/KI control mice (****p < *0.0001*). However, this increase was attenuated in Tau Tg; CRMP2 KI/KI mice (****p < *0.0001*) (Figure S1B).

**Fig. 1:**
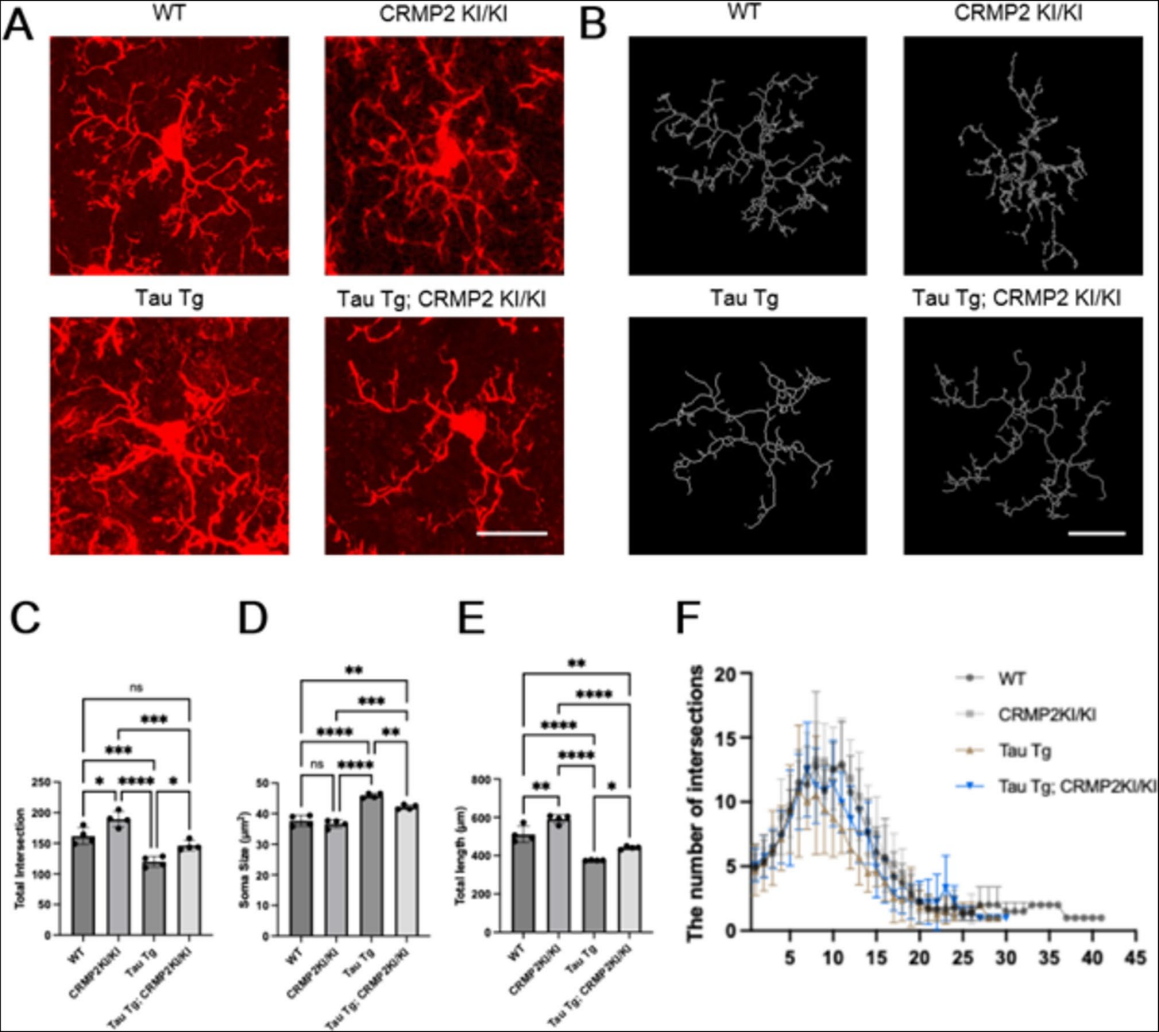
Comparison of Microglial Morphology. **A** Microglia were visualized using IBA1 immunostaining. Images of the hippocampus at 60 × magnification were used for analysis. Scale bar shows 20 μm. **B** Representative images of 3D microglial morphology reconstruction obtained from each group with SNT. **C, D, E**: The increase in microglial soma size in Tau Tg; CRMP2 KI/KI mice was attenuated compared to that in Tau Tg mice. By analyzing the number of microglial intersections and branch length. Analysis of microglial intersections and branch length showed that Tau Tg; CRMP2 KI/KI mice had higher intersection counts and longer branch lengths than Tau Tg mice, but lower than WT mice. Consistently, the reduction in morphological complexity was less pronounced in Tau Tg; CRMP2 KI/KI mice than in Tau Tg mice. WT, n = 4; CRMP2 KI/KI, n = 4; Tau Tg, n = 4; Tau Tg; CRMP2 KI/KI, n = 4. The differences were analyzed using One-Way ANOVA Test followed by Tukey’s test; Data are presented as the mean ± SEM values. (ns, not significant, *p < 0.05, **p < 0.01, ***p < 0.001, ****p < *0.0001*). **F** Sholl analysis confirmed that the reduction in microglial morphological complexity, based on the distribution of intersections at varying lengths, was less pronounced in Tau Tg; CRMP2 KI/KI mice than in Tau Tg mice. WT, n = 4; CRMP2 KI/KI, n = 4; Tau Tg, n = 4; Tau Tg; CRMP2 KI/KI, n = 4. The differences were analyzed using Two-Way ANOVA Test followed by Tukey’s test; Data are presented as the mean ± SEM values. (ns, not significant, *p < 0.05, **p < 0.01, ***p < 0.001, ****p < *0.0001*)

As microglia accumulated in the hippocampus, we continued to observe whether they adopted an activated morphology. Therefore, we analyzed microglial morphology, including soma size, dendritic process length, and branch intersections (Fig. 1A–E). Microglia in Tau Tg mice exhibited soma hypertrophy and a pronounced loss of process complexity compared with WT and CRMP2 KI/KI controls, which had significantly larger soma size, fewer total branch intersections, and shorter overall branch lengths. Microglia from Tau Tg; CRMP2 KI/KI mice displayed a lower activation state than those from Tau Tg mice: soma enlargement was attenuated (**p < *0.01*), and the increases in branch intersections and process length were diminished (*p < *0.1*). Moreover, Sholl analysis showed that at critical radii of 8–12 µm, microglia from Tau Tg; CRMP2 KI/KI mice exhibited intersections that differed from those of microglia from Tau Tg mice (***p < *0.001*), with their branching profile more closely resembling that of WT (**p < *0.01*) (Fig. 1F). Taken together, these findings demonstrate that preventing CRMP2 phosphorylation alleviates tau-induced process atrophy and loss of arborization.

### Attenuated Activation Trends in Astrogliosis by CRMP2 Phosphorylation Inhibition

Brain sections were immunostained with anti‐GFAP as a marker for astrocytes, and the number of GFAP-positive astrocytes was quantified. We also measured astrocyte density in the hippocampi of WT, CRMP2 KI/KI, Tau Tg, and Tau Tg; CRMP2 KI/KI mice (Figure S1A, C). Tau Tg mice showed an increase in astrocyte density compared to WT and CRMP2 KI/KI controls (****p < *0.0001*), but this increase was slightly reduced in Tau Tg; CRMP2 KI/KI mice (****p < *0.0001*). This indicates that CRMP2 phosphorylation influences the tau-driven astrocyte activation.

**Fig. 2:**
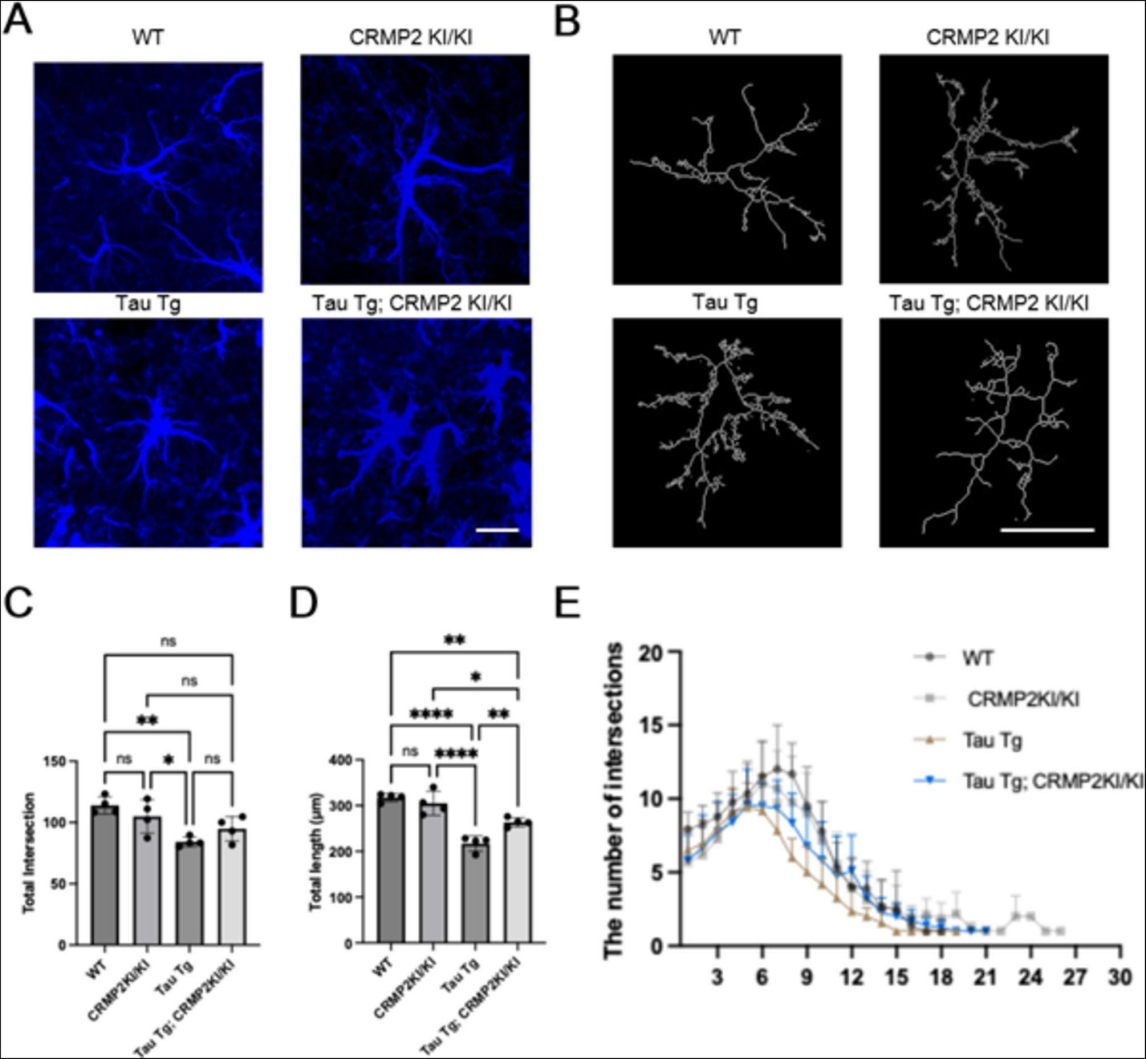
Comparison of Astrocytic Morphology. **A** Astrocytes were visualized by GFAP immunostaining. Imaging taken in the hippocampus at 60 × magnification was analyzed. Scale bar shows 20 μm. **B** Representative images of 3D astrocytic morphology reconstruction obtained from each group with SNT. **C** The increase in astrocytic density in Tau Tg; CRMP2 KI/KI mice was attenuated compared to that in Tau Tg mice. **D, E** Analysis of astrocytic intersections, branch length showed that Tau Tg; CRMP2 KI/KI mice had higher intersection counts and branch lengths than Tau Tg mice but lower than WT and CRMP2 KI/KI mice. The reduction in morphological complexity was also less pronounced in Tau Tg; CRMP2 KI/KI mice than in Tau Tg mice. WT, n = 4; CRMP2 KI/KI, n = 4; Tau Tg, n = 4; Tau Tg; CRMP2 KI/KI, n = 4. The differences were analyzed using One-Way ANOVA Test followed by Tukey’s test; Data are presented as the mean ± SEM values. (ns, not significant, *p < 0.05, **p < 0.01, ***p < 0.001, ****p < *0.0001*). **F** Sholl analysis confirmed that the reduction in astrocytic morphological complexity was less pronounced in Tau Tg; CRMP2 KI/KI mice than in Tau Tg mice. WT, n = 4; CRMP2 KI/KI, n = 4; Tau Tg, n = 4; Tau Tg; CRMP2 KI/KI, n = 4. The differences were analyzed using Two-Way ANOVA Test followed by Tukey’s test; Data are presented as the mean ± SEM values. (ns, not significant, *p < 0.05, **p < 0.01, ***p < 0.001, ****p < *0.0001*)

The process length and number of branch intersections of astrocytes were evaluated to determine astrocytic morphology (Fig. 2A–D). Astrocytes in Tau Tg mice exhibited shorter total process lengths and fewer branch intersections than those in wild-type (WT) and CRMP2 KI/KI mice. In contrast, astrocytes from Tau Tg; CRMP2 KI/KI mice showed a less pronounced loss of morphological complexity than Tau Tg astrocytes, displaying longer processes and more branch intersections.

The Sholl analysis plot compares dendritic complexity among each group by plotting the average number of intersections at successive radial distances from the soma (1–40 µm) (Fig. 2E). Tau Tg neurons show a clear loss of branching, with peak intersections around 6–8 µm falling well below WT levels and remaining reduced across most radii. In contrast, Tau Tg; CRMP2 KI/KI neurons partially recovered from this deficit; their intersection curve rose closer to that of WT (****p < *0.0001*), especially at the critical 6–12 µm range, demonstrating that preventing CRMP2 phosphorylation mitigates Tau-induced dendritic atrophy (***p < *0.001*).

### Inhibition of CRMP2 Phosphorylation by CRMP2 KI Alters Neuronal Cox-2 Levels

Hippocampal CA1 neurons in Tau Tg mice exhibited a significant increase in Cox-2 intensity compared to those in WT and CRMP2 KI/KI controls (****p < *0.0001*) (Figure S2) as previously reported (Yoshiyama et al., 2007). Introducing the CRMP2 KI/KI mutation into the Tau Tg background significantly reduced this upregulation. However, Cox-2 levels in Tau Tg; CRMP2 KI/KI neurons remained elevated compared to those in WT and CRMP2 KI/KI mice. These results suggest that the inhibition of CRMP2 phosphorylation attenuates Tau-driven neuroinflammatory responses.

## Discussion

Tauopathy is a key pathological characteristic of chronic neuroinflammation in AD, and it is essential to study the effects and mechanisms of CRMP2 phosphorylation inhibition in tauopathy models. We previously reported no significant difference of tau and phosphorylated tau distributions in the hippocampus between Tau Tg and Tau Tg; CRMP2KI/KI mice (Nogchi et al., 2025). Given the absence of detectable differences in tau pathology, we concentrated on a detailed analysis of morphological changes in glial cells. We observed that the Tau Tg AD mouse model showed both a higher density and morphological alterations of microglia and astrocytes in the hippocampus compared to the WT and CRMP2 KI/KI controls (Figure S1, 1, 2). Morphological alterations are mainly reflected in increased soma size, shortened process length, and reduced branch intersections, which are characteristic of glial activation (Tao et al., 2022). However, these changes were markedly attenuated in Tau Tg; CRMP2 KI/KI mice (Figs. 1, 2).

Subsequently, we assessed Cox-2 expression in hippocampal neurons to determine whether the suppression of glial activation by introducing CRMP2 KI was more likely attributable to the inhibition of CRMP2 phosphorylation in neurons than in glial cells. We chose Cox-2 because Tau Tg mice show an increase in Cox-2 expression in neurons. This increase was suppressed in PS19; CRMP2 KI/KI mice, although it was still higher than that in WT and CRMP2 KI/KI controls (Figure S2), suggesting that the suppression of CRMP2 phosphorylation in neurons is also involved in this process. These results suggest that the suppression of CRMP2 phosphorylation in neurons is also involved. Our data demonstrate that inhibition of CRMP2 phosphorylation attenuates Tau-driven neuroinflammation.

Our study had several limitations. First, relying solely on morphological changes and Cox-2 expression in hippocampal neurons as readouts provides only a partial view of neuroinflammatory pathways. Future studies will utilize multiplex immunostaining, flow cytometry, or RNA-seq to capture the full spectrum of cytokines, chemokines, and glial subtypes. Second, although we inferred that CRMP2 phosphorylation may improve tauopathy pathology, we have not yet demonstrated direct molecular interactions between CRMP2 and Cox-2 or specific microglial activation factors.

In summary, inhibition of CRMP2 phosphorylation suppressed glial activation in the PS19 mouse model. This suggests that CRMP2 phosphorylation is involved in glial activation induced by the neuronal overexpression of P301S-mutant tau.

## Supplementary Information

Below is the link to the electronic supplementary material.

Supplementary file1 (PDF 337 KB)

## Data Availability

The data that support the findings of this study are available from the corresponding author upon request.

## Abbreviations

Aβ: Amyloid beta
AD: Alzheimer’s disease
Cdk5: Cyclin-dependent kinase 5
CRMP2: Collapsin response mediator protein 2
CA1: Cornu Ammonis 1
MTs: Microtubules
OCT: Optimal cutting temperature
Tg: Transgenic mice
CRMP2KI: CRMP2 S522A Knock-in/Knock-in mice
Cox-2: Cyclooxygenase-2
