# Supplementary material for: Suppression of Glial Activation in Tau Transgenic Mice Through Inhibition of CRMP2 Phosphorylation: a Morphometric Analysis
Source: Neuromolecular Med. 2025 Oct 14;27(1):70. doi: 10.1007/s12017-025-08891-9 (PMC12521301; doi:10.1007/s12017-025-08891-9)
Supplement: Supplementary file 1 — Supplementary file1 (PDF 337 KB) [file 12017_2025_8891_MOESM1_ESM.pdf]

Supplemental Information

Suppression of glial activation in Tau transgenic mice through inhibition of CRMP2 phosphorylation: a morphometric analysis

Wanying Li[1], Toshiki Kubota[1], Valeria Ayala Guevara[1], Yoshio Goshima[2], Takaomi C Saido[3], Toshio Ohshima[1,4]

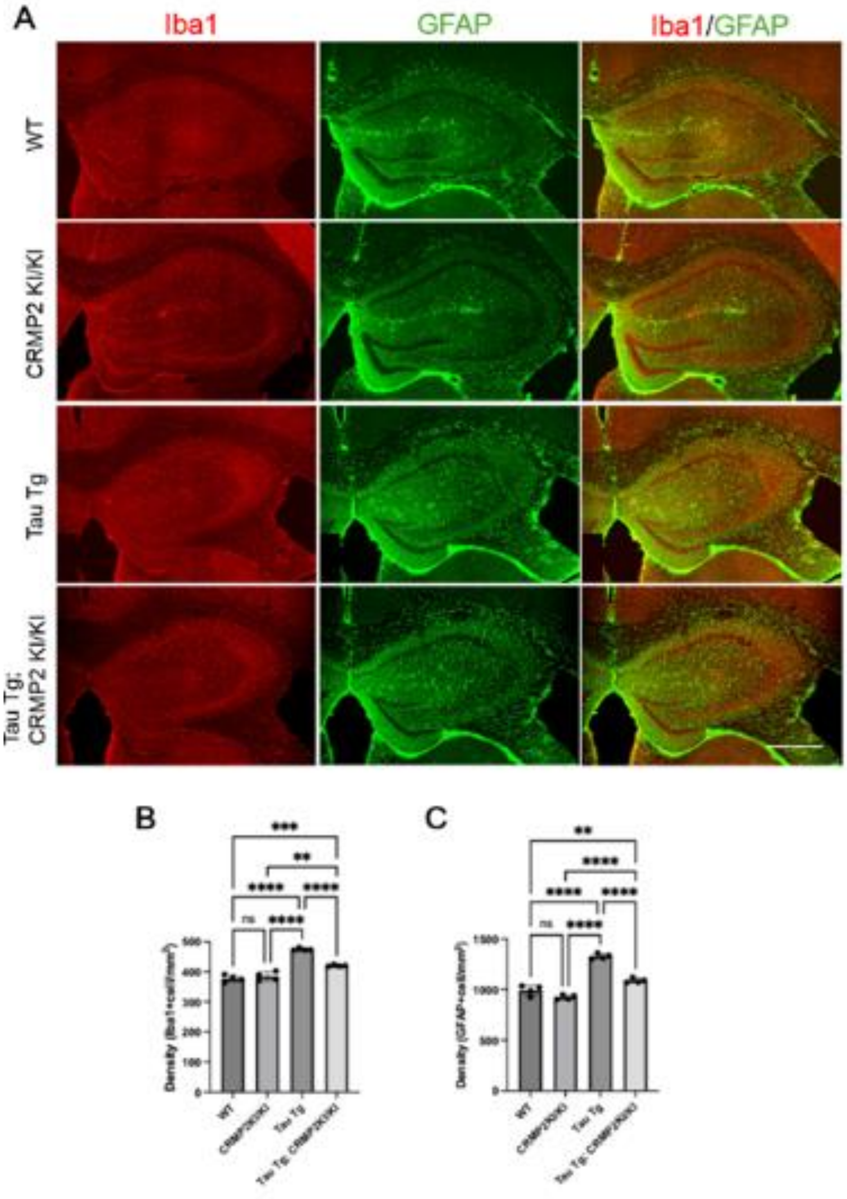

Supplemental Figure 1. Changes in glial dynamics

A: Representative images of Iba1+ microglia and GFAP+ astrocytes in the hippocampus of brain tissue from 5-month-old (5m) mice in each group. Scale bar shows 500 μm.

B: The density of microglia in each group was calculated and compared. The increase in microglial density in Tau Tg; CRMP2KI/KI mice was attenuated compared to that in Tau Tg mice. (Tau Tg; CRMP2KI/KI vs. Tau Tg; ****$p<0.001$).

C: Astrocyte density in each group was calculated and compared. Astrocyte density was significantly elevated in Tau Tg mice, but this elevation was markedly reduced in Tau Tg; CRMP2KI/KI mice (Tau Tg; CRMP2KI/KI vs. Tau Tg; ****$p<0.001$). WT, n=4; CRMP2 KI/KI, n=4; Tau Tg, n=4; Tau Tg; CRMP2 KI/KI, n=4. The differences were analyzed using One-Way ANOVA Test followed by Tukey's test; Data are presented as the mean ± SEM values. (*ns, not significant*, *$p<0.05$, **$p<0.01$, ***$p<0.001$, ****$p<0.0001$).

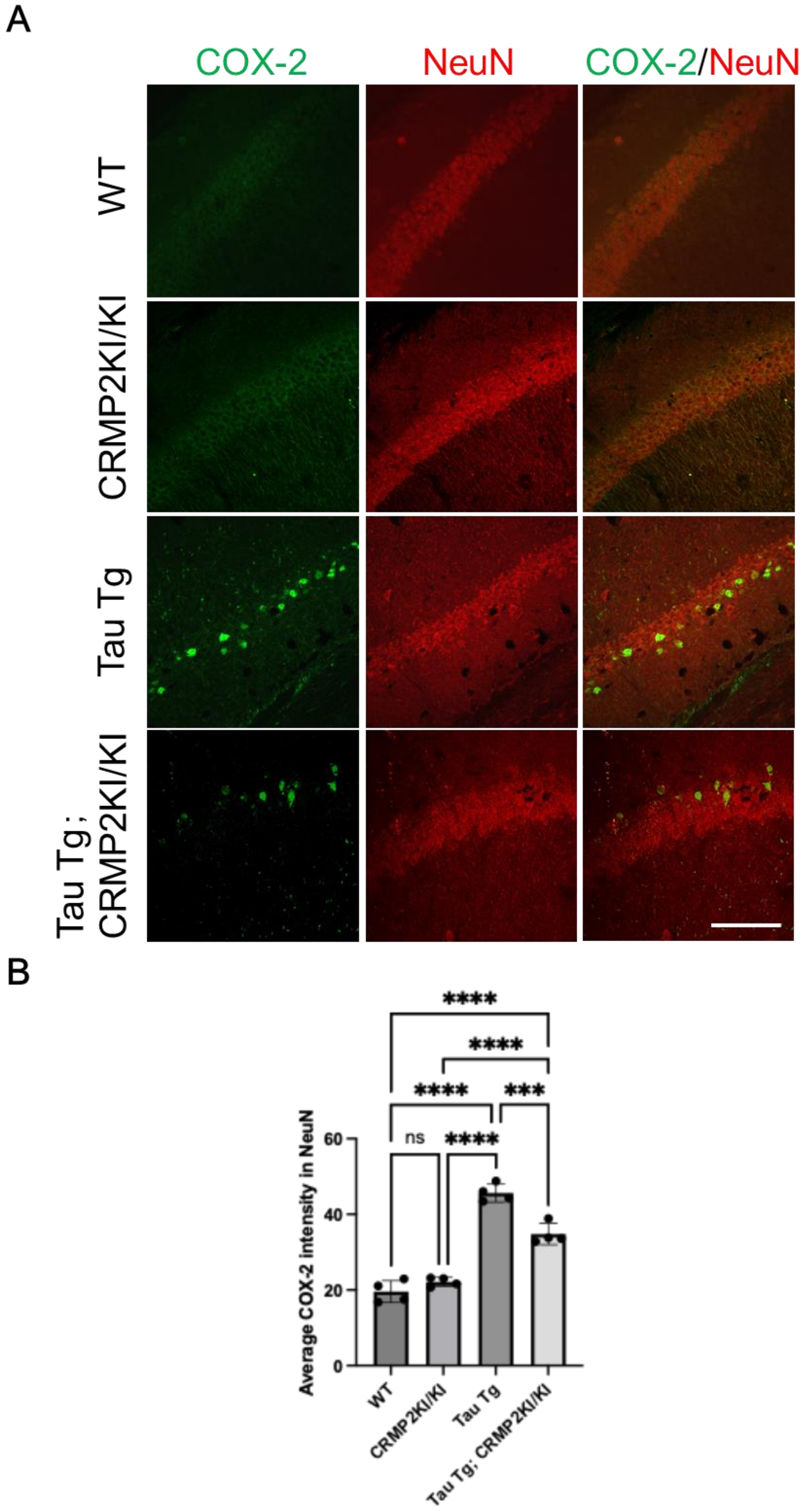
A
COX-2
NeuN
COX-2/NeuN
WT
CRMP2KI/KI
Tau Tg
Tau Tg;
CRMP2KI/KI
B
****
****
****
***
ns
****
Average COX-2 intensity in NeuN
60
40
20
0
WT
CRMP2KI/KI
Tau Tg
Tau Tg; CRMP2KI/KI

Supplemental Figure 2. Assessment of Neuroinflammatory Marker Levels in Hippocampal Neurons

A: The CA1 region of the hippocampus was stained with antibodies against NeuN (red) and Cox-2 (green) in 5-month-old (5m) mice in each group. Scale bar shows 100 μm.

B: Cyclooxygenase-2 (Cox-2) is a marker of neuroinflammation. The expression of Cox-2 in Tau Tg; CRMP2 KI/KI mice was lower than that in Tau Tg mice.

WT, n=4; CRMP2 KI/KI, n=4; Tau Tg, n=4; Tau Tg; CRMP2 KI/KI, n=4. The differences were analyzed using One-Way ANOVA Test followed by Tukey's test; Data are presented as the mean ± SEM values. (*ns, not significant, *p<0.05, **p<0.01, ***p<0.001, ****p<0.0001*).

Materials and Methods

Aminals

Heterozygous P301S tau transgenic mice (PS19 line) (Tg(Prnp-MAPT*P301S)PS19Vle/J , Yoshiyama et al., 2007), originally generated on a mixed genetic background, were backcrossed to C57BL/6 strain (B6) from C3xB6 hybrid and maintained on this background. CRMP2KI/KI (CRMP2$^{S522A/S522A}$) mice, in which the phosphorylation site Ser522 was replaced with Ala, were generated as described previously (Yamashita et al., 2012) and backcrossed to B6 from 129xB6 hybrid. Wild-type (WT) C57BL/6 J mice were used as controls. P301S Tau Tg; CRMP2KI/KI mice were generated as described (Noguchi et al., 2025).

Immunofluorescence

Animals were deeply anesthetized with a triple anesthesia cocktail (medetomidine hydrochloride 0.3 mg/kg, midazolam 4 mg/kg, and butorphanol tartrate 5 mg/kg), and transcardially perfused with 4% paraformaldehyde (PFA) solution in phosphate-buffered saline (PBS). Fixed brains were extracted from the skull and post-fixed overnight in the 4% PFA (4℃ O/N), followed by 30% sucrose solution in PBS for three days. The brains were microtomed (Leica Microsystems, Wetzlar, Germany) into 30 µm slices. After permeabilization for 15 minutes in 0.5% Triton X-100 in PBS, blocking for 1h in 5% bovine serum albumin (BSA), the slices were incubated 4 °C overnight with primary antibodies diluted in PBS. After PBS wash, slices were subsequently incubated with secondary antibodies in PBS at RT 3h. Slices were then mounted on glass slides in Invitrogen™ Fluoromount-G™ Mounting Medium. The following antibodies were used for staining: anti- IBA1 (rabbit; AB_839504, FUJIFILM; 1:400), anti- GFAP (mouse IgG1; A21282, Thermo Fisher Scientific; 1:400), anti- NeuN (rabbit IgG1; 702022, Invitrogen; 1:400), anti- Cox-2 (mouse IgG1, 12-7810, COSMO BIO CO; 1:400), anti- rabbit Alexa 594, and anti- mouse Alexa 488 (Abcam; 1:500 for each).

Image analysis

Fixed tissue was imaged using an FV3000 confocal microscope (Olympus) equipped with a 10× dry objective (N.A. 0.30), a 40× oil-immersion objective (N.A. 1.30), and a 60× oil-immersion objective (N.A. 1.42).

Data analysis

Images were analyzed using ImageJ (National Institutes of Health) software packages.

(1)Density

Microglia in the hippocampus were analyzed in Z-projected images (confocal image: 0.312 μm/pixel, 2 μm Z-step along the rostro-caudal axis, 10 slices, maximum intensity projection). The number of microglia was divided by the hippocampus region to calculate the density.

(2)Soma size

The mean value of the image was adjusted to a radius of 2 pixels. (1024 × 1024 pixels, 0.139 μm/pixel, 2 μm Z-step, 10 slices). Soma areas in the hippocampal region were quantified using the wand tool-Tolerance (1200) in ImageJ.

(3)Morphology

The number of microglial and astrocytic branch intersection and microglial process total lengths were determined from confocal 3D image data (1024 × 1024 pixels, 0.139 μm/pixel, 0.5 μm Z-step, 30 slices). Using the ImageJ plug-in, Simple Neurite Tracer, we then quantified microglial and astrocytic morphology by Sholl analysis based on the reconstructed Z-stacked confocal images. All analyses were performed in the hippocampal region.

(4)COX-2

To correct for non-uniform illumination caused by non-uniform staining, we first performed Gaussian

background subtraction and subtracted the Gaussian-blurred image from the original image.

To calculate the signal intensity of COX-2 in neuron, we first extracted the ROI of neuron from the NeuN channel that are less than the mean + 3 standard deviations and applied this ROI to the COX-2 channel.

We then extracted and statistically analyzed the fluorescence signal intensity of each pixel within this ROI. Images were acquired in the hippocampal CA1 region, and the result for each mouse is the average of 3 slices images at X40 magnification of the staining results.

Statistical Analysis

Statistical analyses were performed using GraphPad Prism 10 software (GraphPad Software Inc., La Jolla, CA). One-way analysis or two-way analysis of variance (ANOVA) with Tukey's post-hoc test was used for multiple comparisons. Data are presented as mean ± standard error of the mean (SEM).
